# Coronal and sagittal spinopelvic alignment in the patients with unilateral developmental dysplasia of the hip: a prospective study

**DOI:** 10.1186/s40001-022-00786-w

**Published:** 2022-08-27

**Authors:** Guangyang Zhang, Mufan Li, Hang Qian, Xu Wang, Xiaoqian Dang, Ruiyu Liu

**Affiliations:** 1 Department of Orthopaedics, The Second Affiliated Hospital of Xi’an Jiaotong University, NO.157, Xiwu Road, Xi’an, Shaanxi Province 710004 People’s Republic of China; 2 Department of Orthopaedics, Chengdu Second People’s Hospital, Chengdu, Sichuan Province 610000 People’s Republic of China

**Keywords:** Developmental dysplasia of the hip, Spinopelvic alignment, Coronal and sagittal plane, Correlation, Low back pain

## Abstract

**Background:**

How the hip dysplasia affects the spinopelvic alignment in developmental dysplasia of the hip (DDH) patients is unclear, but it is an essential part for the management of this disease. This study aimed to investigate the coronal and sagittal spinopelvic alignment and the correlations between the spinopelvic parameters and the extent of hip dysplasia or the low back pain in unilateral DDH patients.

**Methods:**

From September 2016 to March 2021, 22 unilateral patients were enrolled in the DDH group with an average age of 43.6 years and 20 recruited healthy volunteers were assigned to the control group with an average age of 41.4 years. The Cobb angle, seventh cervical vertebra plumbline–central sacral vertical line (C7PL–CSVL), third lumbar vertebra inclination angle (L3IA), pelvic incidence (PI), pelvic tilt (PT), sacral slope (SS), thoracic kyphosis (TK), thoracolumbar kyphosis (TLK) and lumbar lordosis (LL) were measured on the standing anteroposterior and lateral full-length standing spine radiographs. Additionally, the Oswestry Disability Index (ODI) and Japanese Orthopaedic Association Back Pain Evaluation Questionnaire (JOABPEQ) were used to assess the degree of low back pain.

**Results:**

Cobb angle (8.68 ± 6.21° vs. 2.31 ± 0.12°), L3IA (4.80 ± 5.47° vs. 0.83 ± 0.51°), C7PL–CSVL (1.65 ± 1.57 cm vs. 0.48 ± 0.33 cm), PT (15.02 ± 9.55° vs. 9.99 ± 2.97°) and TLK (7.69 ± 6.66° vs. 3.54 ± 1.63°) were significantly larger in DDH patients, whereas LL (37.41 ± 17.17° vs. 48.79 ± 7.75°) was significantly smaller (*P* < 0.05). No correlation was found between significantly different spinopelvic parameters and the extent of dysplasia. Statistical analysis revealed correlations between ODI and Cobb angle (r = 0.59, *P* < 0.01), PT (r = 0.49, *P* = 0.02), TK (r =  −0.46, *P* = 0.03) and TLK (r = 0.44, *P* = 0.04). Correlations between JOABPQE score and the Cobb angle (r = −0.44, *P* = 0.04), L3IA (r = −0.53, *P* = 0.01), PT (r = −0.44, *P* = 0.04), and TK (r = 0.46, *P* = 0.03) were also observed.

**Conclusions:**

Cobb angle, L3IA, C7PL–CSVL in coronal plane and PT, TLK in sagittal plane increased, while LL decreased in unilateral DDH patients. These significantly different spinopelvic parameters have no correlation with the extent of dysplasia. Changes in coronal and sagittal plane including Cobb angle, L3IA, PT, TK and TLK were associated with the low back pain in the patients with unilateral DDH.

**Supplementary Information:**

The online version contains supplementary material available at 10.1186/s40001-022-00786-w.

## Introduction

Developmental dysplasia of the hip (DDH) represents a spectrum of hip disorders and unveils a process of disease ranging from mild hip instability to dislocation [1, 2]. DDH was described early by Palletta in 1820, but its etiology is unknown [3]. Many risk factors for DDH have been identified, including being firstborn, female sex, breech positioning in utero and positive family history [4]. Due to the asymptomatic manifestation of DDH, its true prevalence might be uncertain. A cross-sectional study reported that nearly 1.52% (2.07% for women and 0.75% for men) of the population may be affected by DDH [5]. Significant deformities, such as coxa valga, insufficient coverage of the femoral head, increased femoral anteversion, shallow acetabulum, and shortening of the femoral neck, emerge with the development of DDH and would bring great challenge for the treatment of this disease [6–9].

In the DDH patients, the deformity of the hip has been reported in many studies, but the changes in adjacent joints of the hip were rarely studied. A case–control study found that patients with neglected DDH might develop changes in both knee joints [10]. But the changes of spinopelvic alignment in DDH patients are still unclear, even though the spinopelvic alignment has been reported closely associated with many other diseases and might affect the prognosis of them [11]. It was elucidated that the sagittal spinopelvic alignment was significantly different in ankylosing spondylitis patients with moderate and severe deformities [12]. This abnormal spinopelvic alignment was also found in cerebral palsy patients, and some of the parameters were closely related to the clinical symptoms [13]. Hence, it is assumed that the pelvis asymmetry caused by the dislocated femoral head could result in prolonged limping, finally leading to the impaired spinopelvic alignment in coronal and sagittal plane. In an observational study, the changes of spinopelvic alignment in sagittal plane about DDH patients was reported [14]. Nevertheless, this study mainly concentrated on the Crowe IV type, but neglected the other three Crowe types. Additionally, the coronal spinopelvic alignment and the correlations between parameters and low back pain have not been elucidated. As a result, it is of great significance to analyze the changes in coronal and sagittal spinopelvic alignment and its correlations with low back pain in unilateral DDH patients with all Crowe types.

This study aimed at exploring the changes of coronal and sagittal spinopelvic alignment in the unilateral DDH patients and the relationships between the spinopelvic parameters and the extent of hip dysplasia or the low back pain, which could provide much help for the comprehension of changes in spinopelvic alignment and treatment of spinopelvic malformation in the adult unilateral DDH patients.

## Materials and methods

A total of 22 unilateral DDH patients (9 males and 13 females) were enrolled in the observational study from September 2016 to March 2021. All patients were diagnosed with unilateral DDH, and the exclusion criteria included the following: (1) patients who were unable to accurately communicate about the severity of pain; (2) patients with primary hip or spine deformity; (3) patients with obvious deformity in the knee or ankle; (4) patients who suffered from symptomatic spinal stenosis, lumbar disc herniation and other diseases that could affect spinopelvic alignment; (5) patients who had a prior history of spine or hip surgery, and (6) patients with development deformity or paralysis in the lower limbs. For the recruited volunteers, those who had an abnormal radiogram, such as disc space narrowing or symptoms originating from the spine, were excluded from this present investigation. Altogether, 20 subjects (11 males and 9 females) with no skeleton or muscles paralysis in the lower limbs were included as a control group. All hip dysplasia patients and volunteers underwent the anteroposterior and lateral full-length standing spine plain radiographs with approval by the ethics committee of The Second Affiliated Hospital of Xi’an Jiaotong University, and informed consent was acquired by all individuals.

All unilateral DDH patients were divided into four types according to the Crowe classification system after estimation by measurements on the anteroposterior pelvic radiograph [15]. According to the extent of proximal migration of the femoral head, Crowe I is less than 50 per cent subluxation; Crowe II is 50 to 75 per cent subluxation; Crowe III is 75 to 100 per cent subluxation; and Crowe IV, complete luxation.

### Low back pain assessment

All patients completed a questionnaire for the general information (sex, age, height, weight) and concurrent diseases (cardiovascular disease, pulmonary disease, and other extremity deformities). In addition, the low back pain in patients was also recorded based on the Oswestry Disability Index (ODI) [16] and Japanese Orthopaedic Association Back Pain Evaluation Questionnaire (JOABPEQ) [17]. The ODI was completed by the patient alone, while JOABPEQ was performed by the orthopedic surgeons after the examination and consultation. Patients were excluded if their pain was caused by other diseases or originated from another part of the body, such the ankles or knees.

### Radiological measurement

**Fig. 1:**
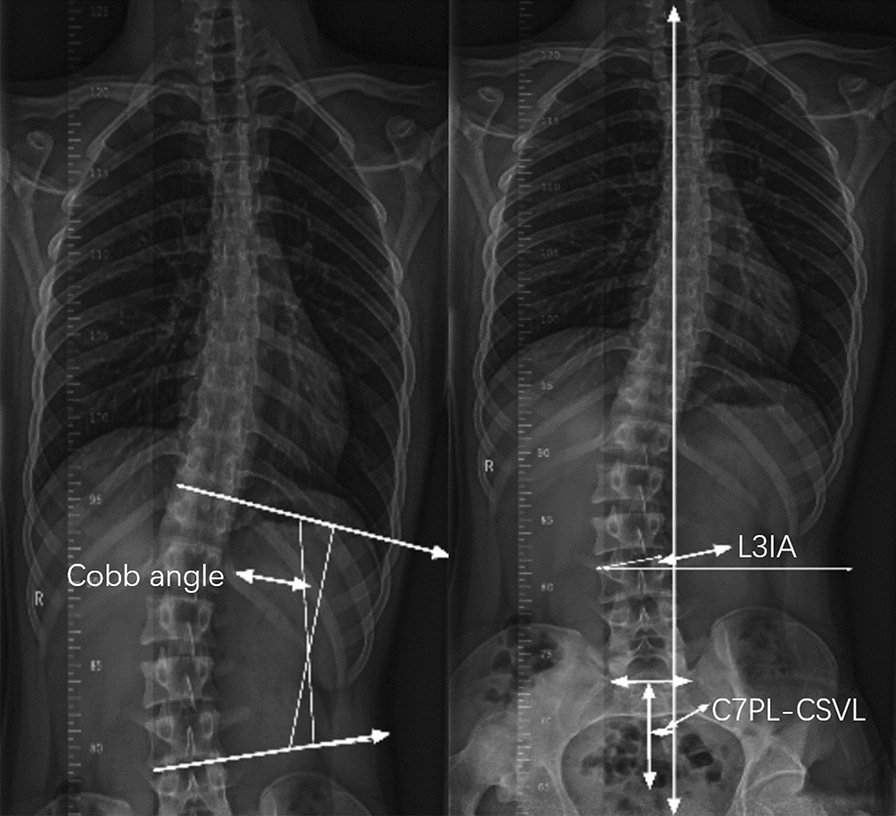
Illustration of the coronal radiographic parameters including Cobb angle, L3IA and C7PL–CSVL. (Cobb angle, the superior surface of the upper vertebra and inferior surface of the lowermost vertebrae; *C7PL–CSVL* seventh cervical vertebra plumbline–central sacral vertical line, horizontal distance traveled by a plumb line dropped from the center of the C7 body to the midperpendicular of S1, *L3IA* third lumbar vertebra inclination angle, the angle between the upper endplate and a horizontal line at L3 body.)

**Fig. 2:**
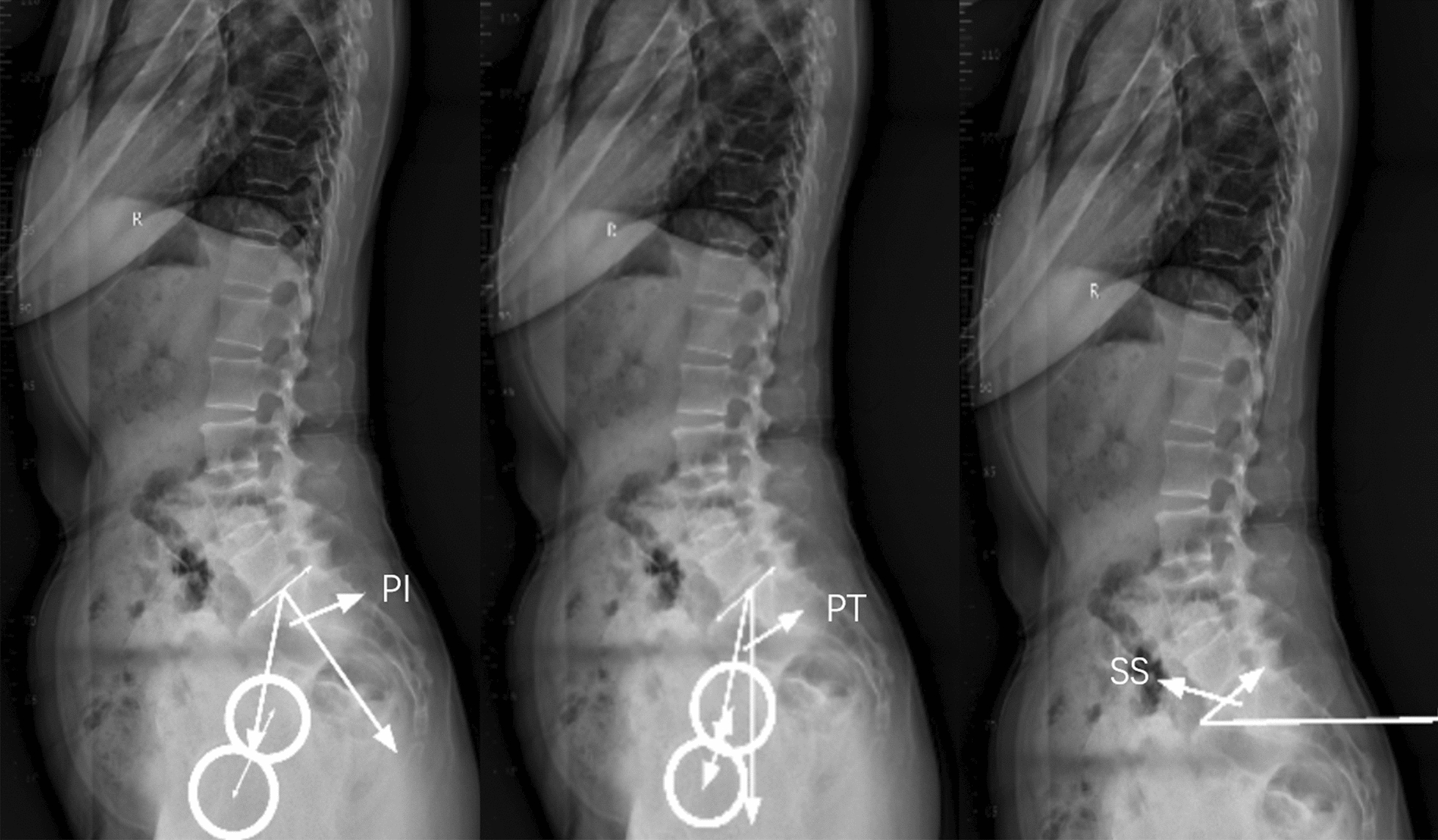
The diagram for the measurement of PI, PT and SS. (*PI* pelvic incidence, angle between a line perpendicular to the sacral end plate and a line joining the middle of the sacral plate and hip axis, *PT* pelvic tilt, between the vertical line and a line joining the middle of the sacral end plate and hip axis,* SS* sacral slope, angle between the sacral end plate and the horizontal line.)

**Fig. 3:**
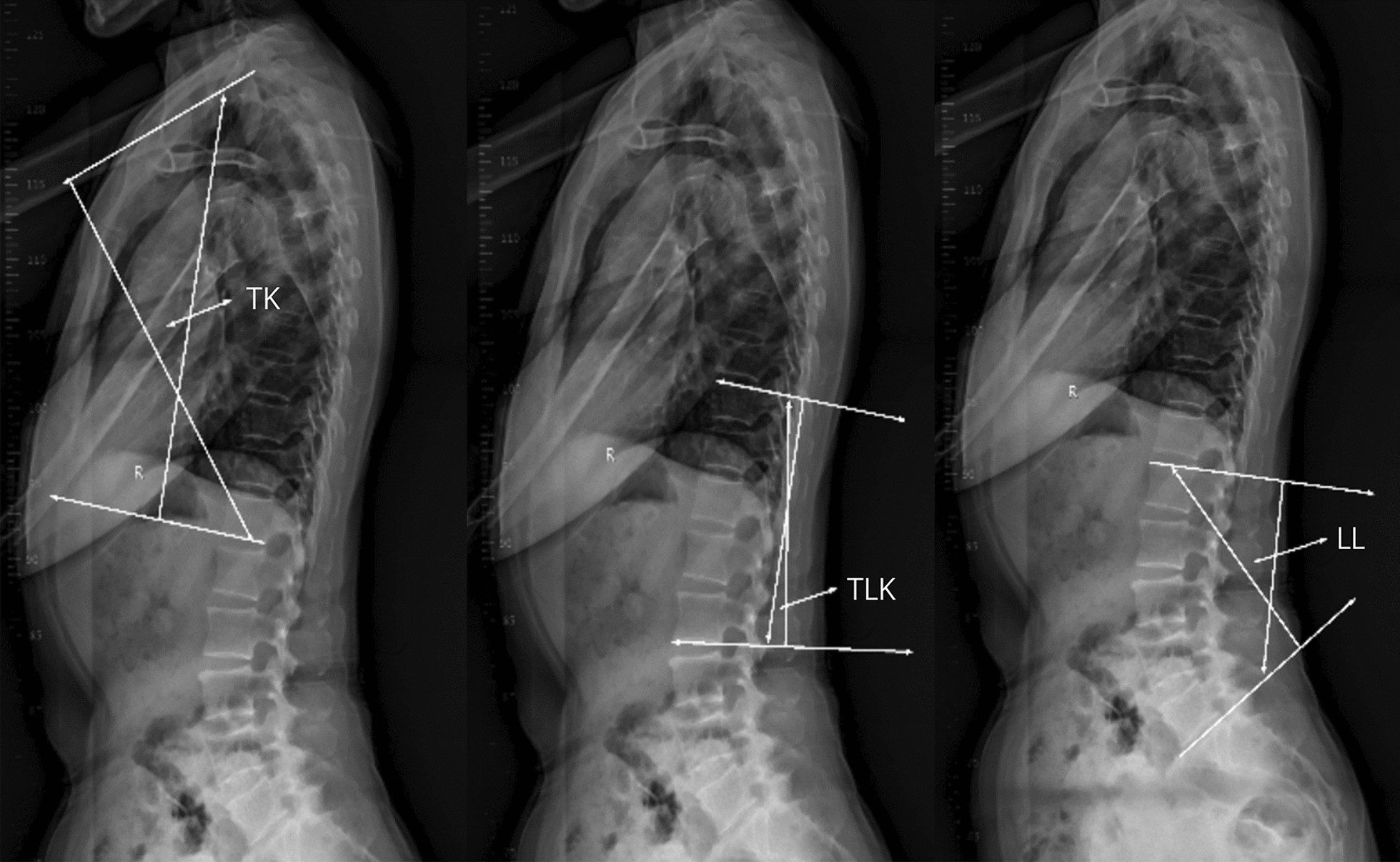
The diagram for the measurement of TK, TLK and LL. (*TK* thoracic kyphosis, angle between the upper end plate of the T2 vertebra and the lower end plate of the T12 vertebra as determined using the Cobb method; *TLK* thoracolumbar kyphosis, angle between the upper end plate of T10 and the lower end plate of L2; *LL* lumbar lordosis, angles measured between the upper end plate of the L1 vertebra and the lower end plate of the S1 vertebra.)

Patients were radiographically evaluated with anteroposterior and lateral full-length standing spine radiographs [18]. In the anteroposterior view, the patient stands in a natural upright position with the knees straight and the arms relaxed. As for the lateral view, the patient also stands in a natural upright position with the knees straight, but the elbows were semi-bent and the hands rested on a support. The coronal spinopelvic parameters including Cobb angle, seventh cervical vertebra plumbline–central sacral vertical line (C7PL–CSVL) and the third lumbar vertebral inclination (L3IA) were measured in the anteroposterior full-length standing spine radiographs (Fig. 1). Cobb angle is calculated by localizing the superior surface of the upper vertebra and inferior surface of the lowermost vertebrae [19]. C7PL–CSVL is defined as horizontal distance traveled by a plumb line dropped from the center of the seventh cervical vertebra (C7) body to the midperpendicular of the first sacral vertebra (S1) [20]. And L3IA is the angle between the upper endplate and a horizontal line at L3 body [21]. In the sagittal plane, the measurement of spinopelvic parameters including pelvic incidence (PI, angle between a line perpendicular to the sacral end plate and a line joining the middle of the sacral plate and hip axis) [22], pelvic tilt (PT, angle between the vertical line and a line joining the middle of the sacral end plate and hip axis) [22], sacral slope (SS, angle between the sacral end plate and the horizontal line) [22] are shown in Fig. 2. And the measurement method of thoracic kyphosis (TK, angle between the upper end plate of T2 and the lower end plate of T12 as determined using the Cobb method) [23], thoracolumbar kyphosis (TLK, angle between the upper end plate of T10 and the lower end plate of L2) [23], lumbar lordosis (LL, angles measured between the upper end plate of L1 and the lower end plate of the S1 vertebra) [24] are illustrated in Fig. 3, all of which were measured in the lateral full-length standing spine radiographs. The measurements of these parameters were recorded digitally with *Image J* (National Institutes of Health, Bethesda, MD, USA) software.

### Statistical analysis

All measurements were performed independently twice with an interval of 2 weeks by two independent orthopedic surgeons to test the reliability. Interclass and intraclass correlation coefficients (ICCs) were used to evaluate the reliability of intra- and inter-group measurements [25]. ICCs values could be categorized as nearly perfect reliability (0.81–1.00), strong reliability (0.61–0.80), moderate reliability (0.41–0.60), fair reliability (0.21–0.40) and poor reliability (0–0.20). The statistical analysis was performed between the DDH patients and the control group using a T test with *SPSS 23.0* software (IBM, Chicago, IL, USA). The Chi-square test was used for the comparison of male/female ratio in the two groups, Pearson correlation and linear regression were performed to analyze correlations between the coronal and sagittal spinopelvic parameters and the extent of hip dysplasia or the low back pain. The results were considered statistically significant at a *P* value < 0.05.

## Results

The characteristics of the unilateral DDH patients and the control group are shown in Table 1. Age, sex, height and weight were not significantly different between the two groups. The extent of hip dislocation was grouped according to the Crowe classification. In the unilateral DDH patients, 8 patients were type I, 5 patients were type II, 5 patients were type III, 4 patients were type IV, and the extent of hip dislocation ranged from 27 to 100% (Table 2). The reliability test showed that the values about intergroup and intragroup ICCs for different parameters were from 0.703 to 0.879, indicting great consistency and credibility of the measurements.

**Table 1:** The basic characteristics of unilateral DDH patients and the control group (mean ± SD)

Parameters	Control	DDH	T/χ^2^	*P*-value
Age (year)	41.4 ± 13.9	43.6 ± 16.0	−0.49	0.63
Male/female (n)	11/9	9/13	0.83	0.36
Height (cm)	161.5 ± 9.2	165.8 ± 12.2	−1.30	0.20
Weight (kg)	72.6 ± 9.8	69.2 ± 8.7	1.18	0.24

*DDH* developmental dysplasia of the hip, *T/χ*^*2*^ T-test or Chi-square.

**Table 2:** The Crowe classification and degree of femoral head subluxation in the unilateral DDH patients

Crowe classification	Percentage of subluxation	N
I	27–47%	8
II	55–75%	5
III	77–96%	5
IV	100%	4

*DDH* developmental dysplasia of the hip; *N* numbers

The coronal and sagittal spinopelvic parameters in unilateral DDH patients and control group are shown in Table 3. In the coronal plane, Cobb angle, L3IA and C7PL–CSVL were larger in the unilateral DDH group. In the sagittal plane, PT and TLK were larger in the unilateral DDH group, LL was smaller in unilateral DDH group than the control group. Further statistical analysis showed there was no correlation between the significantly different coronal and sagittal spinopelvic parameters and the extent of hip dislocation (Additional file 1: Table S1).

**Table 3:** The spinopelvic parameters in unilateral DDH patients and control group (mean ± SD)

Plane	Parameters	Control	DDH	T	*P*-value
Coronal	Cobb angle (°)	2.31 ± 0.12	8.68 ± 6.21	−4.58	< 0.01*
	C7PL–CSVL (mm)	0.48 ± 0.33	1.65 ± 1.57	−3.27	< 0.01*
	L3IA (°)	0.83 ± 0.51	4.80 ± 5.47	−3.23	< 0.01*
Sagittal	PI (°)	51.44 ± 10.98	44.41 ± 14.28	1.78	0.08
	PT (°)	9.99 ± 2.97	15.02 ± 9.55	−2.26	0.03*
	SS (°)	37.70 ± 8.35	39.87 ± 13.43	−0.62	0.54
	TK (°)	33.61 ± 12.03	33.23 ± 12.56	0.10	0.92
	TLK (°)	3.54 ± 1.63	7.69 ± 6.66	−2.71	0.01*
	LL (°)	48.79 ± 7.75	37.41 ± 17.17	2.72	0.01*

The T-test was used to determine the differences between the parameters

*DDH* developmental dysplasia of the hip, *C7PL–CSVL* seventh cervical vertebra plumbline–central sacral vertical line, *L3IA* third lumbar vertebra inclination angle, *PI* pelvic incidence, *PT* pelvic tilt, *SS* sacral slope, *TK* thoracic kyphosis, *TLK* thoracolumbar kyphosis, *LL* lumbar lordosis.

*P < *0.05*

With respect to the degree of low back pain, the Cobb angle in coronal parameters was associated with ODI (r = 0.59, *P* < 0.01) and JOABPEQ score (r = −0.44, *P* = 0.04), and L3IA was related to JOABPEQ score (r = −0.53, *P* = 0.01) in unilateral DDH patients. As for the sagittal parameters, PT (r = 0.49, *P* = 0.02), TK (r =  − 0.46, *P* = 0.03) and TLK (r = 0.44, P = 0.04) were found to be correlated with the ODI, while PT (r = −0.44, *P* = 0.04) and TK (r = 0.46, *P* = 0.03) were also found to be correlated with JOABPEQ score in unilateral DDH patients (Table 4, Additional file 1: Table S2).

**Table 4:** Correlations between the spinopelvic alignment parameters and ODI, JOABPEQ score in unilateral DDH patients

Plane	Parameters	ODI		JOABPEQ	
		**r**	***P***	**r**	***P***
Coronal	Cobb angle (°)	0.59	< 0.01*	−0.44	0.04*
	C7PL–CSVL (mm)	0.16	0.48	−0.35	0.11
	L3IA (°)	0.41	0.06	−0.53	0.01*
Sagittal	PI (°)	−0.03	0.90	0.03	0.91
	PT (°)	0.49	0.02*	−0.44	0.04*
	SS (°)	−0.01	0.98	< 0.01	0.99
	TK (°)	−0.46	0.03*	0.46	0.03*
	TLK (°)	0.44	0.04*	−0.43	0.05
	LL (°)	−0.21	0.35	0.22	0.33

Pearson correlation analysis was used to determine the relationships between the parameters. r, correlation coefficients

*DDH* developmental dysplasia of the hip, *ODI* Oswestry Disability Index, *JOABPEQ* Japanese Orthopaedic Association Back Pain Evaluation Questionnaire, *C7PL–CSVL* seventh cervical vertebra plumbline–central sacral vertical line, *L3IA* third lumbar vertebra inclination angle, *PI* pelvic incidence, *PT* pelvic tilt, SS sacral slope, *TK* thoracic kyphosis, *TLK* thoracolumbar kyphosis, *LL* lumbar lordosis

*P < *0.05*.

## Discussion

Although many studies on orthopedic surgery in patients with DDH have been well performed, the focus of most has been on improvements in the reconstruction of the hip joint [26], while the study about spinopelvic alignment in DDH patients was less. Appreciation of the coronal and sagittal spinopelvic alignment is essential to recognize the adjacent changes resulting from DDH. This study found that Cobb angle, L3IA, C7PL–CSVL, PT and TLK were significantly larger while LL was significantly smaller in unilateral DDH patients compared with the control group. There was no correlation between the significantly different spinopelvic parameters and the extent of hip dysplasia. The statistical analysis revealed correlations between the ODI and the Cobb angle, PT, TK and TLK. Furthermore, correlations were found between JOABPEQ score and the Cobb angle, L3IA, PT and TK.

Developmental dysplasia of the hip accompanies a continuum of deformities that could cause impaired balance in pelvic and spinal. With the help of 3D imaging techniques, Yi-fan et al. illustrated the asymmetric abnormalities of the affected hemipelvis in patients with unilateral Crowe-IV DDH [27]. Spinal deformities in DDH occur secondary to the pelvic imbalances due to postural and muscular forces. It was reported that Crowe IV DDH patients might exhibit abnormal sagittal spinal–pelvic alignment, but the coronal parameters and correlations between these parameters and low back pain have not been investigated [14]. This current study revealed the different coronal and sagittal spinopelvic parameters in unilateral DDH patients of all Crowe types and these differential parameters were not correlated with the extent of hip dysplasia. Moreover, the correlations between coronal and sagittal spinopelvic parameters and low back pain have been well elucidated.

As for the coronal spinopelvic parameters, the Cobb angle and C7PL–CSVL have been regarded as the indicators of coronal balance. In this analysis, the Cobb angle, C7PL–CSVL and L3IA in unilateral DDH patients were all larger than the control group, revealing the existence of scoliosis. Previous studies showed that prolonged lameness might cause functional scoliosis because the lumbar spine had to compensate for pelvic obliquity to maintain balance [28]. And Yu et al. found that sacroiliac joint and spine would work together to achieve coronal balance if the sacroiliac join cannot fully compensate for the imbalance [29]. Hence, it is speculated that unilateral dislocation of the femoral head causes asymmetry of the lower limbs and prolonged lameness, resulting in changes and imbalance in the pelvic structure, eventually leading to imbalance of spine. In a retrospective study, it was found that increased Cobb angle was associated with poor static coronal balance, subsequently leading to the low back pain [30]. It was consistent with our results that Cobb angle positively correlated with the low back pain in this study, which might be explained by the impaired balance in unilateral DDH patients.

Regarding the sagittal spinopelvic parameters, larger PT and smaller LL were observed in the unilateral DDH patients. It was elucidated that the increase in PT compensated for the loss of LL [31]. The individuals had to maintain sagittal balance by extending the hip, which indicated a posterior tilt of the pelvis. However, the compensation mechanism of PT is limited. When the PT can no longer increase, the body experiences decompensation and sagittal imbalance. Therefore, the predisposing factor of increased PT could be the loss of LL. Additionally, Noshchenko et al. revealed that structural characteristics also fundamentally regulate and determine LL [32]. Many factors, such as age-induced degeneration of the intervertebral disc, decrease in the height of the lumbar intervertebral disc, loss of lumbar disc height caused by compression fractures, and source flat deformity, could cause changes in LL. Moreover, the limp caused by lower extremity shortening with the dislocation of the femoral head would change the pelvic structure and imbalance, which might lead to a decrease of LL and an increase in TLK in patients with unilateral DDH. However, the specific mechanism remains unclear.

The spinopelvic parameters have been reported to be correlated with low back pain and the progression of DDH disease [33]. Lafage et al. found that the pelvic position measured via PT affected health-related quality of life in patients [34]. In a retrospective study, pain and reduced function were associated with the global alignment sagittal vertical axis [35]. The SS, PT and PI were regarded as representations of lumbosacral pelvic orientation and PT and TLK have been deemed as delegates of sagittal balance [13]. In this current study, the sagittal parameters including PT, TK and TLK were related to low back pain based on ODI and JOABPEQ score in unilateral DDH patients. These parameters could reflect some structural features of the pelvis and were closely associated with functions, pain, coronal alignment, sagittal alignment and spinopelvic balance. Additionally, the increasing trend of PT and its relationship with rating scale emphasized the importance of PT in orthopedic surgery, underlining the correction of PT was the main goal of spinal orthopedic surgery. For the vertebral physiologic curvature malformation of unilateral DDH patients, the increase in TLK also played a similar role in addition to the reduction in LL and TK. Therefore, we are supposed to concentrate more on the improvement of vertebral physiologic curvature abnormalities to assess the patient's symptoms during the follow-up period of DDH surgery.

The limitations about this present study should also be mentioned. Firstly, it was noticed that the sample size was limited in this study, because all the individuals enrolled should be unilateral DDH patients without intervention, and the subjects who had been treated in childhood were excluded. Secondly, though ages in the DDH patients varied quite a lot and might affect the low back pain, we did not perform subgroup analysis due to the limited sample size. As a result, we would include more individuals to conduct further analysis for the unilateral DDH patients in the future.

## Conclusions

The Cobb angle, C7PL–CSVL, L3IA in the coronal plane and PT, TLK in sagittal plane increased, while LL decreased in the unilateral DDH patients. There was no correlation between the significantly different spinopelvic parameters and extent of hip dysplasia. In addition, the changes of Cobb angle, L3IA, PT, TK and TLK were closely related to the low back pain in the unilateral DDH patients, which could provide much help for the comprehension of changes in spinopelvic alignment and treatment of spinopelvic malformation in the adult unilateral DDH patients.

## Supplementary Information

**Additional file 1: Table S1.** Correlations between significantly different spinopelvic parameters and the extent of hip dysplasia in unilateral DDH patients. **Table S2.** The results of linear regression between spinopelvic parameters and low back pain.

## Data Availability

The data and materials supporting the conclusions are included in this article and supplementary materials.

## Abbreviations

DDH: Developmental dysplasia of the hip
ODI: Oswestry Disability Index
JOABPEQ: Japanese Orthopaedic Association Back Pain Evaluation Questionnaire
C7PL–CSVL: Seventh cervical vertebra plumbline–central sacral vertical line
L3IA: Third lumbar vertebra inclination angle
PI: Pelvic incidence
PT: Pelvic tilt
SS: Sacral slope
TK: Thoracic kyphosis
TLK: Thoracolumbar kyphosis
LL: Lumbar lordosis
ICCs: Inter- and intra-class correlation coefficients
CI: Confidence interval
