# Supplementary material for: Coronal and sagittal spinopelvic alignment in the patients with unilateral developmental dysplasia of the hip: a prospective study
Source: Eur J Med Res. 2022 Aug 27;27:160. doi: 10.1186/s40001-022-00786-w (PMC9419408; doi:10.1186/s40001-022-00786-w)
Supplement: Supplementary file 1 — Additional file 1: Table S1. Correlations between significantly different spinopelvic parameters and the extent of hip dysplasia in unilateral DDH patients. Table S2. The results of linear regression between spinopelvic parameters and low back pain. [file 40001_2022_786_MOESM1_ESM.docx]

**Table S1** Correlations between significantly different spinopelvic parameters and the extent of hip dysplasia in unilateral DDH patients

| Plane | Parameters | Extent of hip dysplasia | |
| --- | --- | --- | --- |
|  |  | **r** | ***P*** |
| Coronal | Cobb angle (°) | -0.241 | 0.281 |
|  | C7PL-CSVL (mm) | -0.200 | 0.372 |
|  | L3IA (°) | -0.243 | 0.276 |
| Sagittal | PT (°) | -0.219 | 0.328 |
|  | TLK (°) | -0.423 | 0.050 |
|  | LL (°) | 0.066 | 0.771 |

DDH: developmental dysplasia of the hip; C7PL-CSVL, seventh cervical vertebra plumbline-central sacral vertical line; L3IA, third lumbar vertebra inclination angle; PT, pelvic tilt; TLK, Thoracolumbar kyphosis; LL, Lumbar lordosis. Pearson correlation analysis was used to determine the relationships between the parameters. r, correlation coefficients; * P＜0.05.

**Table S2.** The results of linear regression between spinopelvic parameters and low back pain

| Marking System | Linear Regression Relationships |
| --- | --- |
| ODI | ODI= 1.1×Cobb+2.66（r= 0.59, *P*＜0.01） |
|  | ODI= 0.59×PT+3.32（r= 0.49, *P*= 0.02） |
|  | ODI= 0.76×TLK+6.35（r= 0.44, *P*= 0.04） |
|  | ODI= -0.42×TK+26.2（r= -0.46, *P*= 0.03） |
| JOABPEQ | JOABPEQ = -0.61×Cobb+23.67（r= -0.44, *P*= 0.04） |
|  | JOABPEQ = -0.82×L3IA+22.36（r= -0.53, *P*= 0.01） |
|  | JOABPEQ = -0.39×PT+24.26（r= -0.44, *P*= 0.04） |
|  | JOABPEQ = 0.31×TK+8.05（r= 0.46, *P*= 0.03） |

ODI: Oswestry Disability Index; JOABPEQ: Japanese Orthopaedic Association Back Pain Evaluation Questionnaire; PT: Pelvic tilt; TK: Thoracic kyphosis; TLK: Thoracolumbar kyphosis; L3IA: third lumbar vertebra inclination angle.
